# Supplementary material for: Cerebral visual impairment: genetic diagnoses and phenotypic associations
Source: J Med Genet. 2024 Mar 8;61(6):605–12. doi: 10.1136/jmg-2023-109670 (PMC11137471; doi:10.1136/jmg-2023-109670)
Supplement: Supplementary data [file jmg-2023-109670supp001.pdf]

SUPPLEMENTARY INFORMATION

Supplementary Table 1: CVI-associated genetic diagnoses in DECIPHER	Page 2
Supplementary Table 2: CVI-associated genetic diagnoses in GEL 100KGP	Page 4
Supplementary Table 3: Literature review for CVI-associated genetic diagnoses present in both 100KGP and DECIPHER datasets	Page 8
Supplementary Table 4: DECIPHER CVI gene set GO Molecular Function pathway enrichments	Page 12
Supplementary Table 5: 100KGP CVI gene set GO Molecular Function pathway enrichments	Page 13
Supplementary Table 6: Combined CVI gene sets GO Molecular Function pathway enrichments	Page 14
Supplementary Table 7: Top 20 HPO terms in DECIPHER CVI group and control group.	Page 15
Supplementary Table 8: Top 20 HPO terms in 100KGP CVI group and control group.	Page 15
Supplementary Figure 1: ShinyGO enrichment networks for DECIPHER control gene sets	Page 16
Supplementary Figure 2: ShinyGO enrichment networks for 100KGP control gene sets	Page 17

Shaw E, *et al. J Med Genet* 2024; 61:605–612. doi: 10.1136/jmg-2023-109670

Supplementary Table 1: CVI-associated genetic diagnoses in DECIPHER

Key
* Genes already known to be associated with CVI on HPO website (https://hpo.jax.org/app/).
# Genes represented in both the DECIPHER and GEL cohorts

Gene	Number of participants with CVI with a pathogenic variant reported	Number of participants with CVI with a likely pathogenic variant reported
GRIN2B*#	2	1
IQSEC2	1	1
SMC1A*	1	1
GRIN1*	0	2
ITPR1	0	2
ARV1	1	0
CACNA1E*	1	0
CASK	1	0
CDKL5*	1	0
CEP290	1	0
CERT1*	1	0
DNM1	1	0
FOXG1 #	1	0
GFM1	1	0
GNAO1	1	0
KCNQ2	1	0
KDM5C	1	0
OPA1	1	0
PLP1	1	0
TCF4*#	1	0
TUBA1A	1	0
ATRX	0	1
DOCK6	0	1
DYNC1H1*	0	1
FARS2	0	1
GRIN2A	0	1
KAT6A*#	0	1
KIF1A*#	0	1
NAA10	0	1
NGLY1	0	1
PAX6	0	1
PDHA1 #	0	1
RARS2 #	0	1

Shaw E, *et al. J Med Genet* 2024; 61:605–612. doi: 10.1136/jmg-2023-109670

SCN1A*	0	1
SLC16A2	0	1
STXBP1*	0	1

Supplementary Table 2: CVI-associated genetic diagnoses in GEL 100KGP

Key
* Genes already known to be associated with CVI on HPO website (https://hpo.jax.org/app/).
# Genes represented in both the DECIPHER and GEL cohorts

Gene Name	Number of participants with CVI with a TIER 1 variant reported	Number of participants with CVI with a TIER 2 variant reported
PITRM1	2	1
KCNT1	2	0
CACNA1A	1	3
SHANK3*	1	3
KANSL1	1	2
GRIN2B*#	1	1
WDR73	1	1
TCF4*#	1	0
TUBB4A	1	0
TUBB2A	1	0
SRD5A3	1	0
HNRNPU	1	0
NR2F1*	1	0
POLR3A	1	0
CSTB	1	0
SIN3A	1	0
GNAS	1	0
HECW2*	1	0
CBL	1	0
MTOR	1	0
SETD2	1	0
SCN2A	1	0
UBA5	1	0
DMD	0	5
MT-ATP6	0	5
HIVEP2*	0	3
TRIO	0	3
CCDC22	0	3
MT-ATP8	0	3
KMT2D	0	3
FLNA	0	3
MT-ND5	0	2
KAT6A*#	0	2
ANKRD11	0	2
KMT2C	0	2
ZIC2*	0	2
KCNQ5*	0	2
CC2D2A*	0	2

COL4A2	0	2
MT-CO1	0	2
NIPBL	0	2
OFD1	0	2
C5orf42	0	2
NF1	0	2
DHTKD1	0	2
MT-CYB	0	1
SLC35A2*	0	1
MCCC1	0	1
EIF2B5	0	1
CHD7	0	1
HCCS	0	1
DDX3X	0	1
FLVCR1	0	1
ZNF711	0	1
ATP13A2	0	1
SZT2	0	1
ABCB7	0	1
ACSL4	0	1
COQ4	0	1
CHD4	0	1
RERE*	0	1
ARID1A	0	1
AUTS2	0	1
PHF8	0	1
KNL1	0	1
CHD8	0	1
SMAD4	0	1
BAG3	0	1
HEPACAM	0	1
TTN	0	1
ALG13	0	1
EBP	0	1
IFIH1	0	1
RNF170	0	1
ATP7B	0	1
SHANK2	0	1
COL11A2	0	1
ACTL6A	0	1
MT-ND1	0	1
BSCL2	0	1
OPHN1	0	1
GK	0	1

ASXL3	0	1
SPG7	0	1
ATM	0	1
NDUFA1	0	1
PUF60	0	1
AFF2	0	1
MTHFR	0	1
ERCC5	0	1
MECP2	0	1
IL1RAPL1	0	1
TREX1	0	1
ACY1	0	1
SLC9A6	0	1
DVL1	0	1
DHX30	0	1
SPTAN1	0	1
PDHA1 #	0	1
AMER1	0	1
UNC80	0	1
FGD1	0	1
MFN2	0	1
DGUOK	0	1
SCAPER	0	1
KIF1A*#	0	1
TSEN2*	0	1
OTC	0	1
MCM3AP	0	1
ATP6AP1	0	1
IKBKG	0	1
SYN1	0	1
PIGA*	0	1
MYT1L*	0	1
CHAMP1*	0	1
TRIP12	0	1
FOXG1 #	0	1
KDM6A	0	1
CACNA1G	0	1
SMARCA2	0	1
FBXO11	0	1
PACS1	0	1
MED13L	0	1
AFF4	0	1
KCNK9	0	1
NPRL3	0	1

WDR45	0	1
ROR2	0	1
CHRNE	0	1
KIF11	0	1
UBE3A	0	1
SMARCC2	0	1
HDAC4	0	1
FAT4	0	1
SACS	0	1
FTCD	0	1
AFG3L2	0	1
PTEN	0	1
RARS2	0	1
RLIM	0	1
TSC2	0	1
WFS1	0	1
ZC4H2	0	1
ARID1B	0	1

Supplementary Table 3: Literature review for CVI-associated genetic diagnoses present in both 100KGP and DECIPHER datasets

Gene	Gene function	Phenotype summary	Case series reference	Total number of patients in paper	Any visual phenotypes?	CVI reported	Other ophthalmological features reported
FOXG1	Transcription factor	Complex neurodevelopmental syndrome, with Rett-like features	[1]	8	yes	8/8 - fatigability of and deficits in visual attention and engagement	
			[2]	122	yes	CVI 41%	Strabismus 64%
			[3]	45	yes	No	Strabismus, poor eye contact, abnormal ocular pursuit<br>16 / 45 as initial concerns<br>38 / 42 on examination
			[4]	26	yes	No	Strabismus, poor eye contact in 9/9 where information available
PDHA1	Pyruvate dehydrogenase deficiency	Mitochondrial dysfunction	[5]	371	yes	No	Optic atrophy 4%<br>Nystagmus 3%<br>Strabismus 1.6%
RARS2	Mitochondrial	Pontocerebellar hypoplasia type 6	[6]	53	Yes	visual impairment unspecified 19%	nystagmus 4%, optic atrophy 4%
GRIN2B	NMDA receptor subunit	Developmental and epileptic encephalopathy, with structural brain malformations	[7]	48 new, 43 from lit	Yes	CVI 7% (a/w cortical malformations)	
TCF4	Transcription factor	Pitt Hopkins Syndrome	[8]	112	yes	No	Strabismus 62%<br>Myopia 48%
			[9]	16	yes	No	Strabismus<br>Myopia<br>Astigmatism

			[10]	10	yes	No	Nystagmus, astigmatism, strabismus, myopia
			[11]	23	yes	No	Myopia<br>strabismus
			[12]	100	yes	No	Up to 50%<br>Strabismus<br>Myopia<br>Astigmatism
KAT6A	histone acetyltransferase, transcriptional regulation	Intellectual disability, Arboleda-Tham syndrome	[13]	5 new, 80 from lit	yes	Visual defects 65%	Strabismus 57%
			[14]	76	Yes	Visual defect 63%	Strabismus 54%
KIF1A	Kinesin motor protein, synaptic vesicle transport	Neurodegeneration and spasticity with or without cerebellar atrophy	[15]	10 new, 99 literature cases	Yes	CVI in 3 / 10 new cases	Optic atrophy common
			[16]	28 new cases	yes		Optic atrophy 25%

1. Boggio EM, Pancrazi L, Gennaro M, et al. Visual impairment in FOXG1-mutated individuals and mice. Neuroscience 2016;**324**:496-508

2. Brimble E, Reyes KG, Kuhathaas K, et al. Expanding genotype-phenotype correlations in FOXG1 syndrome: results from a patient registry. Orphanet J Rare Dis 2023;**18**(1):149

3. Vegas N, Cavallin M, Maillard C, et al. Delineating FOXG1 syndrome: From congenital microcephaly to hyperkinetic encephalopathy. Neurol Genet 2018;**4**(6):e281

4. Kortum F, Das S, Flindt M, et al. The core FOXG1 syndrome phenotype consists of postnatal microcephaly, severe mental retardation, absent language, dyskinesia, and corpus callosum hypogenesis. J Med Genet 2011;**48**(6):396-406

5. Patel KP, O'Brien TW, Subramony SH, et al. The spectrum of pyruvate dehydrogenase complex deficiency: clinical, biochemical and genetic features in 371 patients. Mol Genet Metab 2012;**106**(3):385-94

6. Zhang Y, Yu Y, Zhao X, et al. Novel RARS2 Variants: Updating the Diagnosis and Pathogenesis of Pontocerebellar Hypoplasia Type 6. Pediatr Neurol 2022;**131**:30-41

7. Platzer K, Yuan H, Schutz H, et al. GRIN2B encephalopathy: novel findings on phenotype, variant clustering, functional consequences and treatment aspects. J Med Genet 2017;**54**(7):460-70

8. Whalen S, Heron D, Gaillon T, et al. Novel comprehensive diagnostic strategy in Pitt-Hopkins syndrome: clinical score and further delineation of the TCF4 mutational spectrum. Hum Mutat 2012;**33**(1):64-72

9. Marangi G, Ricciardi S, Orteschi D, et al. The Pitt-Hopkins syndrome: report of 16 new patients and clinical diagnostic criteria. Am J Med Genet A 2011;**155A**(7):1536-45

10. Van Balkom ID, Vuijk PJ, Franssens M, et al. Development, cognition, and behaviour in Pitt-Hopkins syndrome. Dev Med Child Neurol 2012;**54**(10):925-31

11. Goodspeed K, Newsom C, Morris MA, et al. Pitt-Hopkins Syndrome: A Review of Current Literature, Clinical Approach, and 23-Patient Case Series. J Child Neurol 2018;**33**(3):233-44

12. Zollino M, Zweier C, Van Balkom ID, et al. Diagnosis and management in Pitt-Hopkins syndrome: First international consensus statement. Clin Genet 2019;**95**(4):462-78

13. Urreizti R, Lopez-Martin E, Martinez-Monseny A, et al. Five new cases of syndromic intellectual disability due to KAT6A mutations: widening the molecular and clinical spectrum. Orphanet J Rare Dis 2020;**15**(1):44

14. Kennedy J, Goudie D, Blair E, et al. KAT6A Syndrome: genotype-phenotype correlation in 76 patients with pathogenic KAT6A variants. Genet Med 2019;**21**(4):850-60

15. Montenegro-Garreaud X, Hansen AW, Khayat MM, et al. Phenotypic expansion in KIF1A-related dominant disorders: A description of novel variants and review of published cases. Hum Mutat 2020;**41**(12):2094-104

16. Vecchia SD, Tessa A, Dosi C, et al. Monoallelic KIF1A-related disorders: a multicenter cross sectional study and systematic literature review. J Neurol 2022;**269**(1):437-50

Supplementary Table 4: DECIPHER CVI gene set GO Molecular Function pathway enrichments

Enrichment FDR	Number of Genes	Number of Pathway Genes	Fold Enrichment	GO Molecular Function Pathway
6.80E-06	3	5	414.5	Glutamate-gated calcium ion channel activity
1.30E-05	3	8	259	NMDA glutamate receptor activity
1.20E-03	2	11	125.6	Glutamate binding
1.70E-03	2	13	106.3	Glycine binding
8.90E-06	4	28	98.7	Ligand-gated calcium channel activity
2.80E-04	3	27	76.8	Glutamate receptor activity
1.20E-03	3	63	32.9	Transmitter-gated ion channel activity
5.50E-05	5	123	28.1	Calcium channel activity
1.90E-03	3	77	26.9	Extracellular ligand-gated ion channel activity
8.00E-05	5	139	24.8	Calcium ion transmembrane transporter activity
3.60E-03	3	98	21.1	Amyloid-beta binding
8.70E-04	4	148	18.7	Voltage-gated cation channel activity
4.80E-03	3	113	18.3	Neurotransmitter receptor activity
2.80E-04	5	211	16.4	Voltage-gated ion channel activity
2.80E-04	6	350	11.8	Cation channel activity
2.80E-04	6	365	11.4	Gated channel activity
7.90E-04	6	454	9.1	Metal ion transmembrane transporter activity
7.90E-04	6	459	9	Ion channel activity
1.00E-03	6	509	8.1	Channel activity
1.00E-03	6	509	8.1	Passive transmembrane transporter activity
2.60E-03	6	636	6.5	Inorganic cation transmembrane transporter activity
4.80E-03	6	735	5.6	Nucleoside-triphosphatase activity
4.80E-03	8	1364	4.1	Transporter activity

Supplementary Table 5: 100KGP CVI gene set GO Molecular Function pathway enrichments

Enrichment FDR	Number of Genes	Number of Pathway Genes	Fold Enrichment	GO Molecular Function Pathways
7.9E-04	4	29	22.8	Ionotropic glutamate receptor binding
1.2E-03	5	65	12.7	Promoter-specific chromatin binding
2.0E-03	5	74	11.2	Nucleosome binding
1.3E-03	6	108	9.2	Chromatin DNA binding
2.8E-03	7	185	6.3	Helicase activity
1.7E-09	23	617	6.2	Chromatin binding
1.9E-04	11	312	5.8	Transcription coactivator activity
3.9E-04	11	350	5.2	Cation channel activity
3.3E-03	8	255	5.2	Histone binding
1.2E-05	17	550	5.1	Transcription coregulator activity
3.9E-04	12	415	4.8	ATP hydrolysis activity
1.2E-04	16	636	4.2	Inorganic cation transmembrane transporter activity
2.5E-03	11	459	4	Ion channel activity
2.5E-04	16	693	3.8	Cation transmembrane transporter activity
3.9E-04	15	653	3.8	ATP-dependent activity
1.7E-03	13	596	3.6	Transcription factor binding
3.9E-04	16	735	3.6	Nucleoside-triphosphatase activity
7.9E-04	16	798	3.3	Pyrophosphatase activity
7.9E-04	16	802	3.3	Hydrolase activity, acting on acid anhydrides
5.7E-04	18	942	3.2	Zinc ion binding
2.1E-05	27	1446	3.1	Protein-containing complex binding
1.2E-04	23	1248	3	Transmembrane transporter activity
1.4E-04	24	1364	2.9	Transporter activity
2.1E-05	30	1743	2.8	Adenyl nucleotide binding
7.2E-05	28	1662	2.8	ATP binding
1.2E-04	28	1730	2.7	Adenyl ribonucleotide binding
3.3E-03	19	1238	2.5	Transition metal ion binding

Shaw E, *et al. J Med Genet* 2024; 61:605–612. doi: 10.1136/jmg-2023-109670

Supplementary Table 6: Combined CVI gene sets GO Molecular Function pathway enrichments

Enrichment FDR	Number of Genes	Number of Pathway Genes	Fold Enrichment	Pathway
3.95E-05	3	4	102.993976	Mu-type opioid receptor binding
8.41E-05	3	5	82.3951807	Glutamate-gated calcium ion channel activity
3.90E-05	9	148	8.35086291	Voltage-gated cation channel activity
1.62E-05	11	211	7.15913893	Voltage-gated ion channel activity
9.56E-07	16	350	6.27772806	Cation channel activity
3.13E-10	26	617	5.78680362	Chromatin binding
7.01E-05	13	365	4.89103813	Gated channel activity
1.05E-05	16	459	4.7869386	Ion channel activity
5.68E-05	14	415	4.63266076	ATP hydrolysis activity
9.56E-07	21	636	4.53432598	Inorganic cation transmembrane transporter activity
3.14E-05	16	509	4.31670888	Channel activity
3.14E-05	16	509	4.31670888	Passive transmembrane transporter activity
8.66E-07	23	735	4.29725432	Nucleoside-triphosphatase activity
1.98E-05	17	550	4.24460022	Transcription coregulator activity
0.000137495	14	454	4.23470092	Metal ion transmembrane transporter activity
1.74E-06	21	693	4.16137276	Cation transmembrane transporter activity
1.32E-06	23	798	3.9579974	Pyrophosphatase activity
1.32E-06	23	802	3.93825677	Hydrolase activity, acting on acid anhydrides
3.90E-05	18	653	3.78538349	ATP-dependent activity
1.54E-06	29	1248	3.19105267	Transmembrane transporter activity
8.66E-07	33	1446	3.1339799	Protein-containing complex binding
1.23E-06	31	1364	3.12102957	Transporter activity
0.000378354	20	942	2.91561149	Zinc ion binding
9.56E-07	36	1743	2.83632292	Adenyl nucleotide binding
1.71E-06	34	1662	2.80930219	ATP binding
3.86E-06	34	1730	2.69887875	Adenyl ribonucleotide binding

Supplementary Table 7: Top 20 HPO terms in DECIPHER CVI group and control group.

Bold HPO terms= significant after correction for multiple comparisons

	CVI		Control		
Phenotype	CVI Yes	CVI NO	Control Yes	Control No	Fisher's exact test
Global developmental delay	25	36	1050	2527	0.06453865
**Seizure**	18	43	417	3160	**0.00016896**
**Severe global developmental delay**	16	45	314	3263	**0.00006664**
Microcephaly	14	47	516	3061	0.06742103
Generalized hypotonia	8	53	193	3384	0.01777146
**Epileptic spasm**	6	55	27	3550	**0.00001348**
**Generalized-onset seizure**	6	55	76	3501	**0.00225123**
Absent speech	6	55	155	3422	0.05100088
**Agenesis of corpus callosum**	5	56	43	3534	**0.00110523**
**Intellectual disability, profound**	5	56	49	3528	**0.00189006**
Gastroesophageal reflux	5	56	155	3422	0.19126054
**Dystonia**	4	57	31	3546	**0.00254005**
**Generalized myoclonic seizure**	4	57	33	3544	**0.00312485**
Gastrostomy tube feeding in infancy	4	57	37	3540	0.00455815
Progressive microcephaly	4	57	42	3535	0.00690133
Sensorineural hearing impairment	4	57	49	3528	0.01135202
Postnatal microcephaly	4	57	80	3497	0.05104208
2-3 toe syndactyly	4	57	83	3494	0.05676127
Brachycephaly	4	57	116	3461	0.14043867
Intellectual disability	4	57	353	3224	0.51617459

Supplementary Table 8: Top 20 HPO terms in 100KGP CVI group and control group.

Bold HPO terms= significant after correction for multiple comparisons

	CVI Cohort		Control Cohort		
HPO term	Present	Not present	Present	Not present	Fisher's exact test
Global developmental delay	40	31	4797	2747	0.21663523
**Intellectual disability**	30	41	4651	2893	**0.00127693**
**Seizure**	30	41	1151	6393	**0.00000005**
**Delayed speech and language development**	26	45	4661	2883	**0.00002258**
Delayed fine motor development	25	46	3569	3975	0.04295381
Delayed gross motor development	25	46	3907	3637	0.00587709
Generalized hypotonia	19	52	1146	6398	0.01188222
Microcephaly	18	53	1468	6076	0.22779986
**Inability to walk**	15	56	674	6870	**0.00143394**
**Dystonia**	14	57	178	7366	**<0.00000001**
**Gastroesophageal reflux**	12	59	220	7324	**0.00000125**
**Infantile spasms**	12	59	74	7470	**<0.00000001**
**Generalized-onset seizure**	12	59	74	7470	**<0.00000001**
**Intellectual disability, profound**	11	60	67	7477	**<0.00000001**
Morphological central nervous system abnormality	9	62	376	7168	0.00908442
**EEG abnormality**	9	62	54	7490	**0.00000001**
Abnormality of the eye	9	62	801	6743	0.56027663
Failure to thrive	8	63	571	6973	0.25468666
**Epileptic spasm**	8	63	53	7491	**0.00000008**
Abnormality of prenatal development or birth	8	63	247	7297	0.00908442

Shaw E, et al. J Med Genet 2024; 61:605–612. doi: 10.1136/jmg-2023-109670

Supplementary Figure 1: ShinyGO enrichment networks for DECIPHER control gene sets

Networks were produced for 4 out of 10 control gene sets

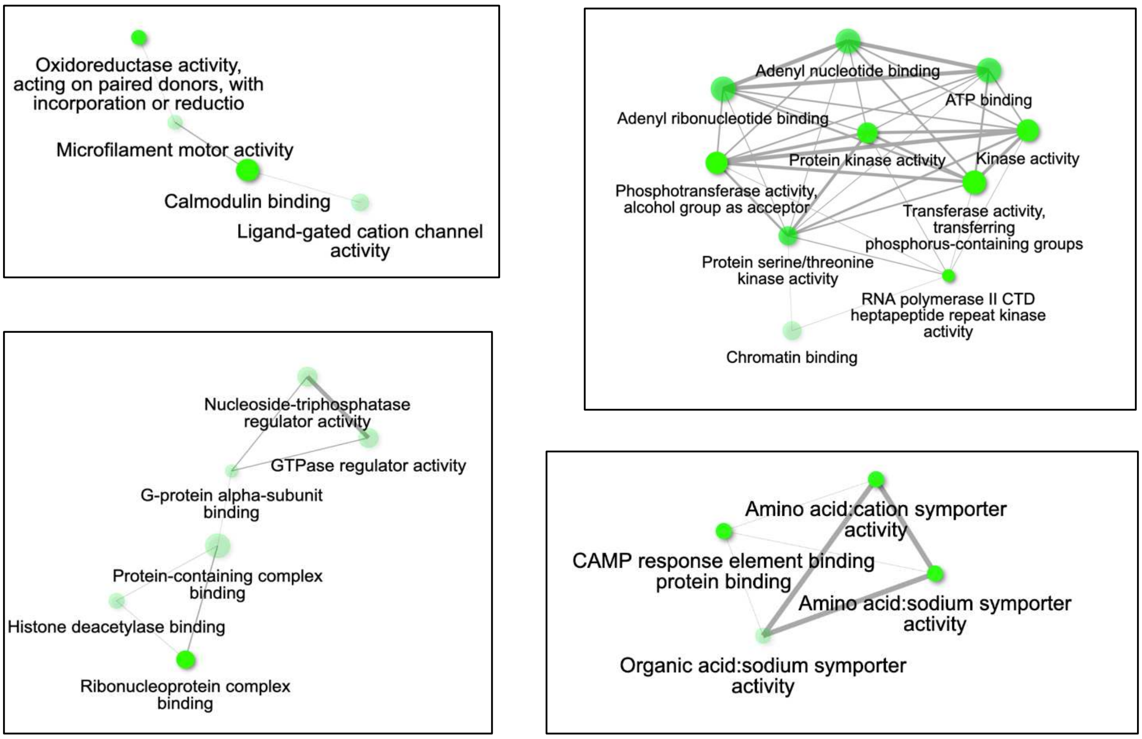

Shaw E, *et al. J Med Genet* 2024; 61:605–612. doi: 10.1136/jmg-2023-109670

Supplemental material BMJ Publishing Group Limited (BMJ) disclaims all liability and responsibility arising from any reliance placed on this supplemental material which has been supplied by the author(s) *J Med Genet*

Supplementary Figure 2: ShinyGO enrichment networks for 100KGP control gene sets

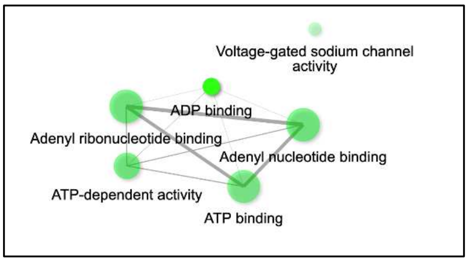

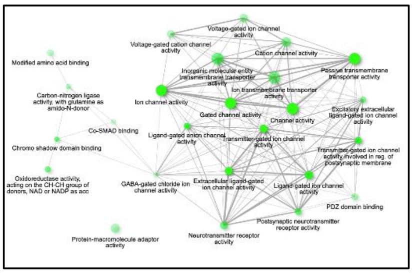

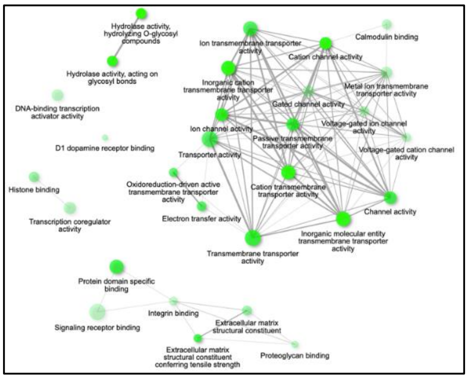

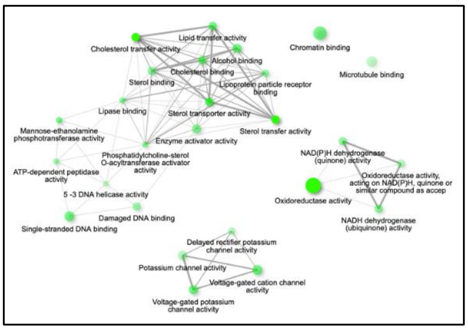

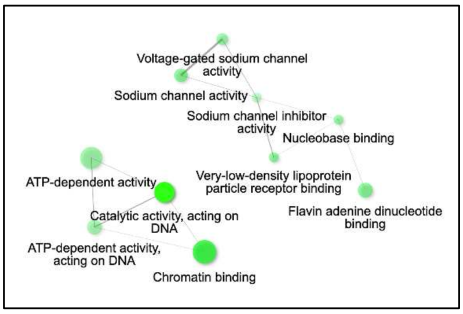

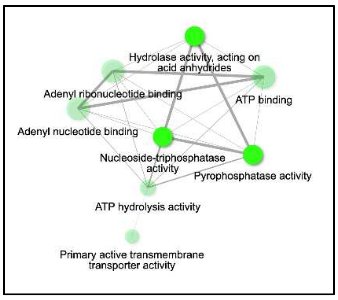

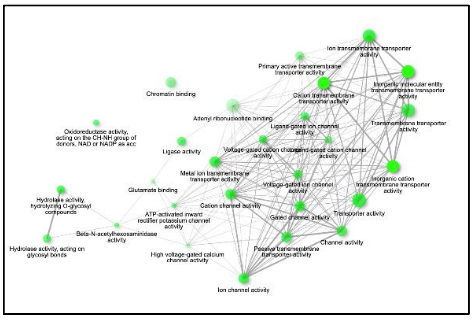

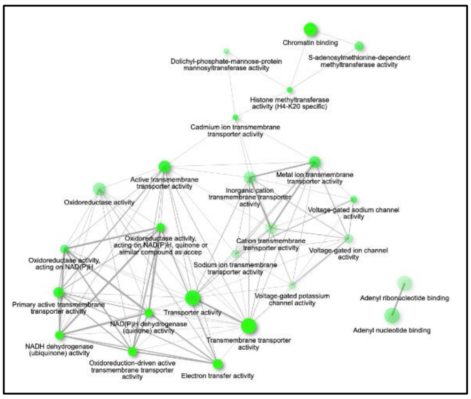

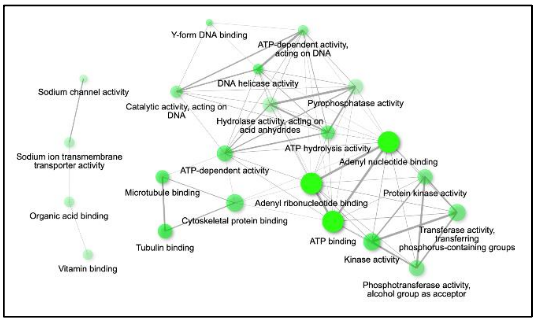

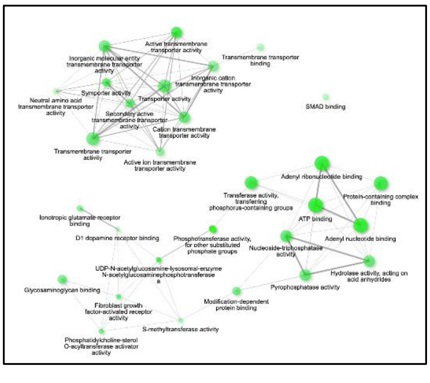

Shaw E, *et al. J Med Genet* 2024; 61:605–612. doi: 10.1136/jmg-2023-109670
